# Licensing Natural Killers for Antiviral Immunity

**DOI:** 10.3390/pathogens10070908

**Published:** 2021-07-19

**Authors:** John M. Cronk, Eleni Fafoutis, Michael G. Brown

**Affiliations:** 1 Department of Microbiology, Immunology, and Cancer Biology, University of Virginia School of Medicine, Charlottesville, VA 22908, USA; jmc5ed@virginia.edu; 2 Human Biology Program, College of Arts and Sciences, University of Virginia, Charlottesville, VA 22908, USA; ecf8b@virginia.edu; 3 Department of Medicine, Division of Nephrology, University of Virginia School of Medicine, Charlottesville, VA 22908, USA

**Keywords:** iKIR, Ly49, self-MHC I, antiviral immunity, polymorphism, missing-self, altered-self

## Abstract

Immunoreceptor tyrosine-based inhibitory motif (ITIM)-bearing receptors (IRs) enable discrimination between self- and non-self molecules on the surface of host target cells. In this regard, they have a vital role in self-tolerance through binding and activating intracellular tyrosine phosphatases which can inhibit cellular activation. Yet, self-MHC class I (MHC I)-specific IRs are versatile in that they can also positively impact lymphocyte functionality, as exemplified by their role in natural killer (NK) cell education, often referred to as ’licensing‘. Recent discoveries using defined mouse models of cytomegalovirus (CMV) infection have revealed that select self-MHC I IRs can increase NK cell antiviral defenses as well, whereas other licensing IRs cannot, or instead impede virus-specific NK responses for reasons that remain poorly understood. This review highlights a role for self-MHC I ‘licensing’ IRs in antiviral immunity, especially in the context of CMV infection, their impact on virus-specific NK cells during acute infection, and their potential to affect viral pathogenesis and disease.

## 1. Introduction

NK cells are essential mediators of host defense against viruses and tumors [1,2]. Patients with NK cell deficiency (NKD) often succumb to overwhelming fatal viral infections before adulthood, and are particularly susceptible to herpesviruses, such as CMV [3]. Importantly, NKD can also affect other innate lymphoid cell (ILC) subsets [4], including tissue-resident ILC1. Although conventional NK cells and ILC1 share the ability to produce IFN-γ, recent murine findings suggest that early ILC1-derived IFN-γ is essential for optimal antiviral immunity [5]. Tissue-resident ILC1 thus likely plays a non-redundant role in innate immunosurveillance (reviewed in [6]). Nonetheless, NK cells are distinctive from tissue-resident ILC1 as they can be recruited to infected tissues where they rapidly limit viral spread by directly lysing infected or malignant target cells [7].

NK cell effector activities are regulated by a diverse array of germline-encoded activation and immunoreceptor tyrosine-based inhibitory motif (ITIM)-bearing receptors (IRs) [1]. Many NK receptors are expressed in a variegated manner, encoded by genes clustered in the natural killer gene complex (NKC) or leukocyte receptor complex (LRC) [8]. Polymorphic Ly49 C-type lectin-like receptors (mouse) and functionally orthologous killer Ig-like (KIR) receptors (human) [9] can either activate or inhibit NK cell functional responses. Whereas NK activation receptor triggering drives signaling cascades [10], IR binding of self-MHC I results in IR clustering and ITIM phosphorylation, promoting recruitment and association with SH2 domain-containing phosphatases (e.g., SHP-1 and SHP-2) [11]. ITIM-activated SHP-1 dominantly impedes stimulatory signals via proximal activation receptors by dephosphorylating Vav, SLP-76, LAT, and PLC-γ [12,13,14]. Self-IR ligation also results in Crk phosphorylation and association with tyrosine kinase c-Abl, which contributes to NK cell inhibition [15]. Phosphorylated Crk is thought to inhibit NK cells by blocking F-actin network formation and constraining activation receptor movement [16].

In addition to their negative regulatory role, IRs can dynamically increase NK cell functionality when they encounter and bind self-MHC I ligands [1,11]. This MHC I-dependent educational process is referred to as ‘licensing’. Relative to unlicensed NK cells, IR-licensed NK cells are more responsive to activation receptor stimulation and thus they can more effectively lyse self-MHC I-deficient (i.e., missing-self) targets [1,11]. Self-MHC I IRs may thus increase activation receptor-mediated recognition of virus-infected target cells. A role for self-MHC I IRs has been difficult to unravel, however, due to their intrinsic ability to license NK effector functions, or dominantly inhibit activation receptor-driven signaling pathways.

Many viruses employ a variety of strategic mechanisms to manipulate MHC I and MHC I-like molecules to evade CD8+ T cell and NK cell immunity (reviewed in [17,18]). Endowed with IRs that can detect missing self-MHC I, licensed NK cells represent a critical arm of the innate immune response to CMVs or other viral pathogens. This review thus examines a role for self-MHC I KIR and Ly49 IRs in antiviral immunity and disease, with a special emphasis on how IR polymorphism differently affects virus-specific NK cell responses.

## 2. Linking Self-Tolerance to NK Cell Functionality

Though well known for their role in self-tolerance through dominant inhibition of cellular activation, self-MHC I-specific IRs help to educate NK cells, thereby maintaining or enhancing effector functions as well [19,20,21]. Compared to NK cells without self-specific IRs, licensed NK cells exhibit greater mTOR/Akt pathway activation at steady-state [22], enhanced expression of adhesion receptors DNAM-1 and LFA-1 [23,24], increased LFA-1-dependent adhesion to target cells [25], and specialized compartmentalization and mobilization of activation receptors in the plasma membrane [26,27]. Enhanced activation of glycolysis and greater accumulation of large granzyme-B filled secretory lysosomes further contributes to heightened proliferative and cytolytic responses of IR-licensed NK cells [28,29]. Self-specific IRs have also been shown to positively regulate type I IFN production in plasmacytoid dendritic cells (pDCs) upon TLR9 stimulation [30]. TLR9-triggered self-IR+ pDCs further exhibit increased lysosomal integrity and stability, which is associated with protection from pyroptosis-like cell death [31]. These findings indicate that IRs can modify the intrinsic cytolytic potential of NK cells by affecting diverse cellular processes. Yet, the molecular pathway by which IR signals license NK cells has not been fully elucidated.

Although a mechanistic basis for licensing remains to be uncovered, several studies have advanced our understanding of the requirements for IR-dependent tuning of NK cell reactivity. A functional IR ITIM domain is essential to bind and activate SH2 domain-containing phosphatases [32], and to sustain the licensing status of NK cells [19,33]. The strength of the inhibitory signals propagated by a given IR/self-MHC I ligand pair differently calibrate the functional responsiveness of licensed NK cells [34], and when transferred into a disparate MHC I environment, mature NK cells become ‘re-educated’ by resetting IR-defined self-tolerance in balance with their potential for activation receptor signaling [35,36]. IR ITIM signaling thus serves to dynamically tune activation receptor signaling pathways during and after NK cell development to promote cellular immunity.

SHP-1 activity is required in licensing since SHP-1-deficient NK cells fail to reject MHC I-deficient tumors and poorly respond to activation receptor crosslinking [22,37]. Interestingly, SHP-2-deficient NK cells exhibit reduced ERK1/2 phosphorylation in response to IL-15 [38]. Because SHP-2 can also bind phosphorylated ITIMs, an IR-ITIM—SHP-2 signaling axis might be important in regulating ERK1/2 signaling pathway in NK cells. In contrast to SHP-1 and SHP-2, SHIP1 has not been shown to directly interact with KIR ITIMs during KIR-mediated inhibition [39,40]. Nonetheless, SHIP1 may play a role in NK cell licensing, as SHIP1-deficient NK cells exhibit reduced stimulation in response to platebound activation receptor ligation, and further fail to mediate acute MHC I-mismatched bone marrow graft rejection [41]. Further investigation thus is needed to more fully elucidate how the balance of SHP-1, SHP-2, and SHIP1 signaling may affect licensed NK cell effector activities and immunity.

ITIM signals drive phosphatase activity and also enhance the functionality of licensed NK cells. One explanation that could account for these seemingly paradoxical observations is that ITIM-regulated signaling molecules are spatially and temporally poised to promote cellular activation during release of inhibition. In this regard, an IR SHP-1 signaling axis leading to Crk phosphorylation may be an important mechanism by which self-MHC I-specific NK cell IRs modulate the movement and spatial distribution of activation receptors in the NK cell plasma membrane [16,26,27,42]. Modulation of E3-ubiquitin ligase c-Cbl phosphorylation may be another molecular pathway by which IR/SHP-1 activity tunes NK cell activation receptor signaling [43]. Alternatively, IRs might promote NK cell functionality by preventing induction of hyporesponsiveness downstream of chronic activation receptor stimulation [21]. If so, it will be important to define which ITIM-regulated signaling molecules downstream of activation receptors (e.g., Vav, SLP-76, LAT, or PLC-γ) are responsible for tolerizing NK cells to activation receptor stimulation. The advent of inducible systems to delete or overexpress the aforementioned signaling molecules in mature primary NK cells will be useful to address these gaps in knowledge.

## 3. Self-KIR IR and HLA Class I Associations with Human Viral Diseases

The effects of IR-dependent tuning of NK cell functionality described above likely regulate the quality and magnitude of NK cell-mediated antiviral immunity in both humans and mice. Moreover, human genetic variation within these diverse NK cell recognition receptors and their polymorphic MHC I ligands may influence the immune response to particular pathogens. Indeed, discrete inhibitory *KIR* (*iKIR*)/*HLA* gene pairs have been associated with disease outcomes in several human chronic viral infections, including human immunodeficiency virus (HIV), hepatitis C virus (HCV), and CMV [44,45].

Genetic association studies of individuals with HIV infection were among the first to identify a role for *iKIR/HLA* gene pairs in antiviral immunity. Notably, the HIV-encoded immunoevasin Nef selectively downregulates HLA-A and HLA-B cell surface expression to evade T cell recognition [46]. HIV-infected targets thus are resistant to missing-self recognition by NK cells expressing HLA-C-specific IRs [46]. However, NK cells lacking HLA-C-specific IRs readily lyse infected cells when the HIV strain is capable of downregulating MHC I [47]. These findings hinted that additional NK IRs may detect HIV-induced alterations in MHC I expression.

Indeed, HIV-infected patient outcomes can be related to HLA-B-binding *KIR3DL1* allotypes. High KIR3DL1 cell surface expression in combination with HLA-B*57 bearing a *Bw4* motif with Ile at position 80 (*Bw4-80I*) corresponds to delayed progression to acquired immune deficiency syndrome (AIDS) in comparison to patients lacking HLA-B*57 [48]. Related to this, licensed NK cells encoding high surface expression *KIR3DL1* and *HLA-B* allotypes exhibit higher reactivity against autologous HIV-infected CD4+ T cells in vitro relative to licensed NK cells encoding low receptor/ligand surface expression *KIR3DL1/HLA-B* allotypes [49]. Thus, akin to murine studies showing greater inhibitory signaling input increases the licensing status of NK cells [34], greater KIR3DL1 and HLA-B density may similarly enhance NK cell cytolytic potential and detection of HIV infection.

Beyond surface expression differences, KIR3DL1 structural polymorphism may also regulate NK functionality through binding or clustering interactions with HLA-B. KIR3DL1 alleles differing in affinity or specificity for HLA-B were shown to differently affect NK cytotoxicity towards HLA-Bw4+ target cells [50]. Moreover, a *KIR3DL1* (Val^47^) allele variant was shown to be significantly associated with elite virus control in HIV-infected HLA-B*57+ patients [51]. HIV seropositive ’elite controllers‘ maintain low, or in some cases undetectable, plasma HIV RNA in the absence of anti-retroviral therapy, and do not progress to AIDS [52]. Hence, self-MHC I-specific iKIR polymorphisms may modify iKIR/HLA binding interactions so that NK cells differently respond to HIV-modified HLA class I proteins, resulting in long-term HIV immunity.

Whereas Nef targets HLA-A and HLA-B to subvert T cell recognition, the HIV-encoded Vpu immunoevasin specifically mediates HLA-C downregulation and limits in vitro HLA-C restricted T cell responses to HIV-infected CD4+ T cells [53]. In support of a role for licensed KIR2DL+ NK cells in missing-self recognition, KIR2DL+ NK cells readily degranulate in response to HLA-C downregulation during coculture with HIV-infected CD4+ T cells [54]. However, licensed KIR2DL+ primary NK cells exhibit impaired ability to inhibit HIV replication relative to their unlicensed KIR2DL+ counterparts, perhaps due to binding of residual HLA-C molecules on the surface of autologous HIV-infected cells [54]. Thus, the above findings indicate HIV immunity may be positively or negatively affected by licensed IR+ NK cells, and patient outcomes may be defined by polymorphisms in iKIRs or their HLA class I ligands which can affect their binding interactions.

Associations between *iKIR*/*HLA* gene pairs and disease outcome have also been observed in hepatitis C virus (HCV) infection. Homozygosity for certain *KIR2DL3* and *HLA-C1* allotypes is associated with resolution of HCV infection in a cohort of individuals exposed to low infectious doses [55]. A separate study revealed that the combination of *KIR3DL1* and an *HLA-Bw4* allele with threonine at position 80 (*HLA-Bw4-80T*) is significantly enriched in seropositive-HCV RNA-negative individuals in comparison to seropositive-HCV RNA-positive individuals [56]. Intriguingly, both KIR3DL1 and KIR2DL3 exhibit peptide selectivity [57,58]. Moreover, KIR2DL3 has been shown to differently bind HCV core-derived peptides presented by a particular HLA-C1 allotype [59]. While a basis for defined iKIR/HLA contributions in HCV control remains to be elucidated, distinct licensed iKIR+ NK cells may be differently responsive to HCV targets.

Similar to HIV and HCV, herpesviruses such as CMV have evolved an arsenal of immunoevasins to evade detection by cytolytic effector cells of the immune system, namely CD8+ T cells and NK cells. Several of the proteins employed by herpesviruses regulate cell surface expression of MHC I or MHC I-like molecules in infected cells, which underscores the importance of the IR/MHC I recognition axis for control of viral spread [17,60]. Despite the fact that all classes of herpesviruses downregulate MHC I [61], licensed iKIR+ NK cells specifically expand in response to CMV infection in comparison to Epstein-Barr virus (EBV), Herpes simplex virus (HSV)-1, HSV-2, or Varicella zoster virus (VZV) infection [62], resulting in long-term CMV-associated imprints on the human KIR repertoire.

A direct role for NK cells in antiviral immunity to CMV is supported by a study showing that a T-B+NK+ severe combined immunodeficiency (SCID) patient could control CMV infection in the absence of T cell immunity [63]. Notably, NK cells derived from the patient during peak CMV infection were >80% positive for KIR2DL2/2DS2/2DL3 [63]. A separate study of hematopoietic cell transplant (HCT) recipients showed that licensed KIR2DL3+ NK cells selectively expand following CMV reactivation, which further corresponds with the heightened functionality of self-specific KIR+ NK cells relative to non-self-specific KIR+ NK cells analyzed in this work [64].

The significance of the expansion of licensed NK cells in response to CMV remains unclear. Certain licensed iKIR+ NK cell subsets may be more responsive to CMV reactivation, enabling efficient control of CMV spread. In support of this possibility, *KIR2DL3/2DS2+/HLA-C1*+ CMV seronegative solid organ transplant (SOT) recipients receiving organs from CMV seropositive *HLA-C1*+ donors experience significantly decreased hazard of CMV viremia [65]. Notably, lack of *HLA-C1* expression in *KIR2DL3/2DS2+* SOT recipients or donors is associated with increased risk of CMV viremia [65], suggesting that KIR2DL3/HLA-C1 interactions may be protective during infection. In future studies, it will be of interest to delineate a basis for expansion of licensed KIR2DL3+ NK cells during CMV infection, and the significance of this NK cell subset with respect to limiting CMV spread.

Taken together, the above findings suggest self-specific iKIR/HLA interactions may endow NK cells with increased responsiveness during HIV, HCV, or CMV infection, perhaps by enabling detection of virus-modified self-MHC I. Additional studies are needed to define whether particular iKIR/HLA gene pairs also affect disease risk associations in other viral infections, especially for viruses known to modify MHC I expression or conformation. In this regard, the severe acute respiratory syndrome coronavirus 2 (SARS-CoV-2)-encoded immunoevasin ORF8 was recently shown to re-direct MHC I to lysosomes for degradation [66].

## 4. Bridging Education, Natural Killing, and Antiviral Immunity

IRs that can efficiently distinguish self-MHC I from virus-induced self-MHC I alterations or virus-encoded MHC I mimics may enable more effective NK cell responses and viral clearance. Herein, we highlight murine CMV (MCMV) infection studies in mouse models demonstrating the importance of self-MHC I-specific IRs in antiviral immunity. These studies reveal a combined effect of polymorphism in both MHC I and Ly49 receptors on NK cell antiviral immunity which hints that similar mechanisms may underlie iKIR/HLA associations with human disease.

Prior work using MA/My or C57L-derived mouse strains expressing the MHC I D^k^ protein found that NK cells expressing the D^k^-specific-Ly49G2 inhibitory receptor undergo selective expansion during acute MCMV infection [67,68,69]. This expansion is genetically determined by defined MHC class I (i.e., H-2D^k^) and Ly49G2 IR alleles. Both D^k^-binding MA/My (G2^M^) and C57L (G2^L^) Ly49G2 IRs can license NK cells and enable selective NK expansion in D^k^-bearing host mice during MCMV infection [67,70,71], whereas the C57BL/6 (G2^B6^) IR which does not bind D^k^ cannot [72]. Depletion of G2+ NK cells from MA/My or C57L-D^k^ mice before infection thus abolishes MCMV control [67,68,69,73]. Moreover, because both MA/My and C57L NKC-*Ly49* haplotypes lack a *Ly49h* gene, MHC I-dependent MCMV resistance in these strains is fundamentally distinct from NK-mediated MCMV control in B6 mice.

To further elucidate G2^L^’s role in MCMV immunity, we crossed the C57L NKC (NKC^L^) and D^k^ onto the B6 background. We further used CRISPR/Cas9 editing to ablate Ly49G2 from B6.NKC^L^-D^k^ mice, generating Ly49G2^null^ mice. We discovered G2^null^ mice exhibit significantly increased mortality during MCMV infection, despite an otherwise intact Ly49 repertoire and normal NK cell development [71]. MCMV control in this model is T cell-independent since depletion of CD4+ and CD8+ T cells prior to infection has no effect, and it requires perforin-dependent cytotoxicity (data not shown). Less effective G2^null^ NK activation, differentiation, or proliferation in comparison to wild-type NK cells thus suggests G2^L^ recognition of MCMV targets is vital to deliver highly efficient antiviral immunity.

Parikh et al. have since shown the G2^B6^ or Ly49A^B6^ IRs can likewise enhance NK-mediated antiviral immunity in D^d^-transgenic B6 mice during MCMV infection [74]. Both IRs bind D^d^, license NK cells, and promote rejection of missing-self targets [19,33,75,76]. A functional ITIM was shown essential to trigger licensed IR+ NK control of MCMV in this model system [74], suggesting that the activation of key intracellular tyrosine phosphatases is required in MHC I-dependent antiviral immunity through NK cells [16,68]. Presumably, IR/phosphatase interactions do not propagate inhibitory signals upon interaction with missing-self targets. However, it is possible IR/phosphatase activities could enhance licensed NK responsiveness towards MCMV-induced changes in cell surface MHC I.

A requirement for G2^L^, G2^B6^, or Ly49A^B6^ self-specific IRs in NK-mediated MCMV control suggests each may have a key role in recognition of viral targets, possibly via detection of missing-self MHC I MCMV targets. However, this model does not readily explain why NK cells bearing other D^k^-specific (e.g., Ly49O/V [77]) or licensing (e.g., NKG2A [78]) IRs in G2^null^ mice do not similarly recognize missing-self targets and mediate MCMV control. Hence, select self-IRs may be better equipped to recognize missing-self MCMV targets than others. Alternatively, beyond detection of missing-self cues, perhaps certain self-IRs can distinguish self- from virus-modified MHC I molecules (i.e., altered-self recognition).

Unlike the self-MHC I licensing IRs described above, the H-2K^b^-specific Ly49C IR was shown to impede virus-specific NK cell immunity driven by the Ly49H activation receptor [79], which binds the MHC I-like viral protein m157 [80]. This result is intriguing since K^b^-licensed Ly49C+ NK cells exhibit high basal reactivity at steady-state, efficiently reject missing-self MHC I targets [22,78], and because K^b^ is efficiently downregulated by viral immunoevasins gp40 and gp48 in MCMV-infected cells [81,82]. Whether Ly49C’s failure to release Ly49H+ NK cells to kill MCMV targets is related to its affinity for K^b^ [83], its broad specificity for different MHC I molecules [76], or binding to the MCMV immunoevasin gp34 plus K^b^ (i.e., gp34/K^b^ complexes) is unknown. Nonetheless, polymorphic IRs with differing affinities or specificities for MHC I thus may differently recognize virus-induced changes in cognate self-MHC I expression or conformation on infected targets.

A clue to understanding their role in MCMV immunity was revealed in a study from Babić et al. showing that licensed IR+ NK cells in BALB/c (H-2^d^) mice can control an attenuated MCMV strain lacking the *m04* gene which encodes gp34 [84]. Because gp34 binds nascent MHC I molecules for transport to the cell surface where it facilitates MHC I binding to self-MHC I-specific IRs expressed by NK cells, it can prevent missing-self target recognition [84]. Hence, IR+ NK cell immunity in BALB/c mice is thwarted by gp34 during infection with wild-type MCMV [84]. Licensing IRs thus are exploited by MCMV to prevent NK-mediated clearance of infection.

Licensed NK cell-mediated antiviral immunity may be particularly relevant in the setting of hematopoietic stem cell transplant (HSCT). Transplant recipients frequently undergo pharmacological immunosuppression to prevent complications associated with transplant rejection such as graft versus host disease [85]. However, treatment regimens can leave recipients susceptible to latent virus reactivation, as often occurs when recipients or donors have experienced prior CMV infection. Ensuring optimal reactivity of licensed NK cells in these settings is thus an important consideration. Related to this, it will be important to understand how licensed NK cell functionality is calibrated by MHC I molecules expressed by specific donor- and host-derived cell types.

Evaluation of reconstituted Ly49G2+ NK cells in radiation bone marrow chimeras demonstrated that D^k^ expression in both hematopoietic and non-hematopoietic cells is required for optimal NK cell stimulatory activity, rejection of missing-self targets, as well as efficient control of MCMV infection [70,73]. In support of this, the absence of MHC I ligands on non-hematopoietic cells in irradiation chimeras renders NK cells hyporesponsive to activation receptor stimuli and further tolerizes NK cells to missing-self targets during MCMV infection [86]. Both hematopoietic and non-hematopoietic cell types thus affect the calibration of licensed NK cell functionality and their capacity to mediate antiviral immunity. Altogether these findings further suggest that licensed NK cells are vital antiviral effectors following HSCT. In line with this idea, licensed NK cells selectively proliferate following MCMV infection in syngeneic or allogeneic HSCT recipient B6 mice, as well as D^d^-transgenic NKC^B6^ mice [87,88]. Selective expansion of licensed NK cells in these settings corresponds with greater IFN-γ production and enhanced MCMV control by licensed NK cells [87,88]. Further study is needed to address whether licensed NK cells may affect CMV reactivation in patients following HSCT.

## 5. Selective Activation of Licensed NK Cells during Viral Infection

Whether self-specific IRs recognize missing- or altered-self MHC I, the signal-driving mediators of MHC I-dependent NK cell antiviral immunity are less well defined. Many types of viruses target MHC I antigen processing and presentation pathways to reduce presentation of viral antigens [17]. Yet, certain IRs expressed by NK cells exhibit peptide selectivity, suggesting they may play a key role in pathogen recognition. For instance, the mouse Ly49I IR can distinguish peptides presented by K^d^ tetramers [76]. Similarly, select peptide variants presented by K^b^ molecules modify the strength of Ly49C inhibitory signals [89]. In humans, the KIR3DL1 IR differently inhibits NK clones depending on the peptides presented by HLA-B [57]. Likewise, certain peptides presented by HLA-C weakly bind KIR2DL2/KIR2DL3 and antagonize inhibitory signals delivered by the respective IRs [58]. Self-MHC I-specific IR recognition of an altered peptide repertoire during viral infection thus might facilitate highly efficient NK recognition of viral targets, while also protecting uninfected host cells.

Self-specific KIR activation receptors have similarly been shown to exhibit selectivity for pathogen-derived peptides. For example, KIR2DS2 discriminates HLA-C-presented flaviviral peptides [90]. Moreover, KIR2DS4 was found to specifically recognize conserved bacterial RecA peptides presented by HLA-C [91]. Perhaps related to triggering of activating KIR, peptide variants loaded into the MHC I peptide binding groove can alter the conformational flexibility of MHC I molecules [92,93,94]. These findings beg the question whether certain self-specific Ly49 and KIR are sensitive to viral peptide/protein-associated changes in MHC I conformation during infection.

**Figure 1:**
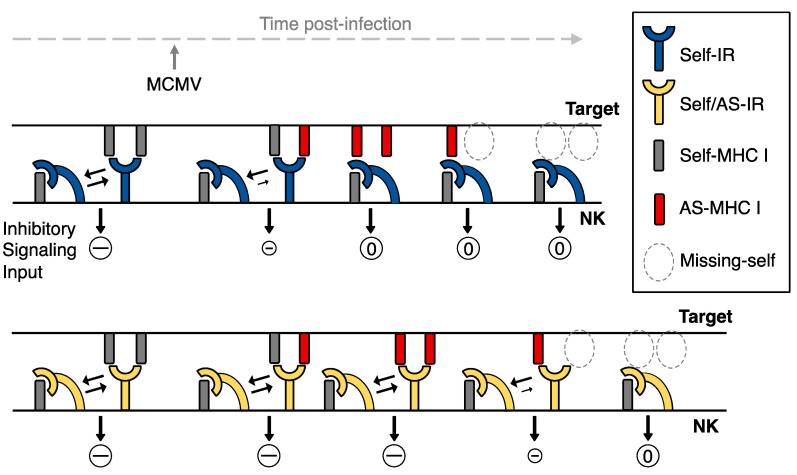
A model for IR-licensed NK sensing of virus infected cells. At steady-state, an equilibrium defines the number of Ly49 IRs engaged in *cis* or in *trans* with self-MHC I molecules and IR negative signaling (–) in NK cells contributes to self-tolerance. Over the course of viral infection, altered-self (AS) MHC I molecules may accumulate at the target cell surface, along with downregulation of surface MHC I (i.e., missing-self). For IRs that are highly specific for self-MHC I relative to altered-self, *cis*-binding may prevail during interaction with infected targets, thereby diminishing negative signaling (0) so that NK cells bearing activation receptors for ligands on infected targets are triggered. However, IRs that can bind self and AS MHC I may continue to dominantly block activation receptor signaling pathways. Only a sufficient loss of self-MHC I then can trigger both types of IRs. Hence, distinct IRs for self-MHC I, like their activation receptor counterparts, may differ in NK sensing of virus infected targets.

Because Ly49 IRs bind self-MHC I on targets (in *trans*) and on the same cell (in *cis*), when NK cells encounter healthy cells an equilibrium (i.e., *cis* ⇋ *trans*) describing IR—self-MHC I interactions thus exists (reviewed in [95]; and see Figure 1). IR binding to *trans* MHC I leads to recruitment and activation of tyrosine phosphatases (e.g., SHP-1) that dominantly turn off activation receptor signals and prevent NK effector functions. A shift in this equilibrium so that *cis* binding prevails upon encountering missing-self MHC I targets, however, results in reduced IR access to the immune synapse in *trans* [96], less inhibitory signaling [97], and increased lysis of missing-self targets [98] that also display ligands for NK activation receptors (Figure 1). Hence, IR polymorphisms that merely bind self-MHC I, or those that bind altered-self MHC I with less affinity than self, should release inhibition and unleash signaling through activation receptors. Licensing IRs which can efficiently discriminate missing- or altered-self MHC I ligands thus may allow highly sensitive NK sensing and recognition of viral targets.

Prior studies suggest Ly49P and Ly49R activation receptors contribute to MCMV control in MA/My mice, whereas the Ly49L activation receptor is linked with MCMV control in BALB.K mice [71,99,100,101]. Ly49P and Ly49L activation receptors both bind gp34-bound MHC I proteins displayed on MCMV targets [99,100,101]. While Ly49R binds D^k^ itself [71,77], it is unclear if it also binds gp34-D^k^ or another MCMV-modified D^k^ ligand. Still, the Ly49R-specific mAb 12A8 given to mice before infection abolishes D^k^-licensed Ly49G2+ NK antiviral immunity during MCMV infection [71]. Activation receptor binding to gp34-MHC I complexes on MCMV targets thus may be analogous to Ly49H+ NK recognition of m157-bearing MCMV targets in B6 mice [80]. Nonetheless, none of these activation receptors have yet been proven essential in MCMV control [65,87,88,89].

Whereas blockade of the NKG2D activation receptor abrogates licensed NK cell immunity in BALB/c or BALB.K mice infected with attenuated Δm04/gp34 MCMV [84], licensed NK cells do not require DAP10 or DAP12 adaptor signaling to control MCMV in D^d^-transgenic B6 mice [74]. Because DAP10 and DAP12 signaling adaptors are used by the Ly49 activation receptors mentioned above as well as NKG2D, these data suggest other stimulatory receptor/adaptor pairs must be involved. While distinct mechanisms appear to be at work, licensed NK cell virus immunity in the different model systems may be similar or partially overlapping. Relatedly, although Ly49R-neutralization may thwart G2^L^ NK cells in MCMV-infected D^k^ mice by blocking an interaction between the activation receptor and its ligand on infected targets, this treatment might instead alter the *cis* ⇋ *trans* equilibrium for G2 binding with D^k^ described in Figure 1 since Ly49R can also bind self-D^k^ proteins in *cis* and *trans* [71]. Usage of multiple different activation receptors by virus-specific licensed NK cells thus might obscure a strict requirement for DAP10/DAP12 signaling.

There is evidence to suggest that similar mechanisms of IR-licensed or Ly49H-like NK cell activation occur in response to viral infection in humans. IR-licensed human NK cells coexpressing the CD94/NKG2C heterodimeric activation receptor clonally expand in CMV-seropositive individuals [62]. Notably, expansion of NK cells expressing self-specific iKIR is most striking for the NKG2C+ subset relative to the NKG2C- subset [62]. Related to this, NKG2C+ NK cells from CMV-seropositive donors selectively proliferate during in vitro coculture with HLA-E expressing target cells [62] as well as CMV-infected fibroblasts [102,103]. Collectively, these data suggest that NKG2C contributes to CMV-specific activation of iKIR-licensed human NK cells.

CMV UL40 peptides were shown to elicit potent degranulation and cytokine production by NKC2C+ ‘adaptive’ NK cells derived from CMV-seropositive patients [104]. In vitro activation of adaptive NKG2C+ NK cells by UL40 peptides was shown to be HLA-E- and CD94-dependent [104]. Moreover, NKG2C+ NK cells from CMV-seronegative donors, but not NKG2C- NK cells, selectively proliferate in vitro in response to UL40 peptide variation [104]. Therefore, akin to Ly49H, the NKG2C activation receptor likely contributes activation of NK cells via direct recognition of CMV-infected targets.

Intriguingly, enhanced proliferation of NKG2C+ NK cells in response to disparate UL40 peptides coincides with increased accumulation of NKG2C+ NK cells co-expressing self-specific iKIR [104]. However, the extent to which IR-licensed human NK cells rely on NKG2C signaling for antiviral immunity to CMV remains unclear. iKIR+ NK cells expand in NKG2C-deficient CMV-seropositive donors, and functionally resemble adaptive iKIR+ NKG2C+ NK cells [105,106]. With the exception of triggering through activation receptors CD16 [107] or Ly49H [108], NK cells require synergistic stimulation via multiple activation receptors (e.g., NKG2D, 2B4, DNAM-1, CD2) to induce cytotoxicity [11]. Certain licensing IRs may lessen this requirement for synergy by regulating the inhibitory activity of ubiquitin ligase c-Cbl [15,109]. An interesting question is whether synergy exists between such licensing IRs and activation receptors. Related to this, expression of activation/adhesion receptor DNAM-1 is strongly correlated with IR licensing of both mouse and human NK cells [23,24]. In humans, DNAM-1 blockade diminishes NK cell degranulation in response to CMV-infected monocyte-derived DCs and HIV-infected CD4+ T cells in vitro [110,111]. DNAM-1 has also been implicated in MCMV-specific unlicensed NK cell expansion in B6 mice [112]. However, licensed murine NK cells lacking DNAM-1 still mediate missing-self lysis of targets [24]. A specific role for DNAM-1 in CMV-specific activation of licensed NK cells thus remains to be elucidated.

## 6. Conclusions

With the seminal discovery that select IRs confer essential MCMV control, now there is evidence that licensed NK cells play a key role in antiviral immunity, though not all IRs are created equal. This distinction is almost certainly linked to IR polymorphism which affects MHC I protein binding and IR-dependent tuning of licensed NK cell effector functions, including functions conserved in mice and humans. Nonetheless, it remains unknown why only certain licensing IRs adequately respond to missing-self MHC I cues during wild-type or attenuated MCMV infection and contribute to viral control. Whether this ability is related to a particular IR’s affinity for MHC I, its ability to license or tune NK effector functions, or its capacity to efficiently detect altered-self MHC I proteins remains an open question. Notwithstanding, multiple virus-responsive activation receptors are rendered impotent in the absence of these vital licensing IRs during MCMV infection. These findings are suggestive that *iKIR/HLA* class I associations in human patients with chronic virus infections may likewise be related to highly efficient NK sensing via licensing IRs. Given their role in self-tolerance, we envision licensed IR-dependent antiviral immunity might also promote adaptive immunity through better overall protection of host cells, lymphoid structures, antigen presenting cells and the development of highly specific T cells and B cells. Resolving these questions may facilitate tailor-made strategies to enhance NK cell cytotoxicity against specific viruses and tumors which downregulate MHC I molecules to evade T cell immunity.

## Data Availability

Not applicable.
